# Controversies in the Treatment Strategies of Intertrochanteric Fractures: A Scoping Review and Discussion of a Literature-Based Algorithm

**DOI:** 10.3390/jcm14072200

**Published:** 2025-03-24

**Authors:** Tilman Graulich, Mohamed Omar, Stephan Sehmisch, Emmanouil Liodakis

**Affiliations:** 1 Department of Trauma Surgery, Hannover Medical School, Carl-Neuberg Str. 1, 30625 Hannover, Germany; omar.mohamed@mh-hannover.de (M.O.); sehmisch.stephan@mh-hannover.de (S.S.); 2 Department of Trauma, Hand and Reconstructive Surgery, Departments and Institutes of Surgery, Saarland University, 66123 Homburg, Germany; emmanouil.liodakis@uks.eu

**Keywords:** intertrochanteric fractures, reconstruction, arthroplasty, complication, algorithm

## Abstract

Intertrochanteric fractures become more and more relevant in an aging population. Despite significant progress in the treatment of these fractures, some technical details, concerning the surgical procedure, are still a matter of strong debate. In this review of the literature, we have included the best evidence available from the last decade in an effort to shed light on some of the most controversial subjects related to intertrochanteric fractures: Treatment in the case of polytrauma or monotrauma? Reconstruction or arthroplasty? Open or closed reduction? Reconstruction with or without additional cables and plates? Cephalomedullary nail or dynamic hip screw (DHS)? Long cephalomedullary nail or short cephalomedullary nail? The results of this scoping review are controversial. By introducing a new therapeutic algorithm, we do not intend to present a new finished guideline but rather arouse a controversial debate about a relevant aspect in geriatric traumatology. These conflicting results are an indication that larger and more well-conducted, high-quality trials are needed in order to gain more secure answers.

## 1. Introduction

Proximal femoral fractures represent an increasing proportion of fractures in an aging society. An increase of 7% was observed in Great Britain between 2020 and 2023, and in Germany we are also seeing a significant increase in these fractures due to the changing social structure (UK’s National Hip Fracture Database (NHFD) [1]. In 2019, they accounted for around 150,000 cases, or 22% of all fractures in Germany [2]. Political efforts are also leading to an increasing focus on level-one trauma centers, as data from colleagues from Mainz show an increase of 100% from 200 to 400 annual cases between 2016 and 2022 [2]. The relevance of these injuries for patients is great, as these are life-threatening injuries, also due to secondary consequences, which are associated with a mortality rate of 5–10% in the first month and 33% in the first year after injury. More than 10% are subsequently unable to return to their previous environment [3,4]. In general, the rule of thirds is used, which states that approximately one-third of injured patients regain their previous level of activity, approximately one-third of patients do not regain their previous level of activity, and approximately one-third of injured patients die within the first year.

### General Considerations for Decision-Making

General considerations for determining the treatment strategies for these fractures include the assessment of the fracture morphology, with the question of mechanical stability, but also the general pattern of injury, i.e., whether it was part of a polytrauma or an isolated trauma. The age and comorbidities of the patients, the functional demands of the patients, and the degree of pre-existing arthrosis are also relevant. Fischer et al. state a rather simplified algorithm, which recommends treating intertrochanteric fractures with a short cephalomedullary nail and subtrochanteric fractures with a long cephalomedullary nail [5]. This algorithm contains essential aspects in the decision-making process for fracture treatment of intertrochanteric femoral fractures and forms the basis of the care structure in many clinics. However, recent studies show that a more differentiated algorithm is needed, and several open questions and controversies remain unclear. The aim of this study was not to conduct a systematic review that strictly follows the PRISMA regulations (Preferred Reporting Items for Systematic review and MetaAnalysis), but rather to examine and discuss controversies.

Finally, an attempt is made to present an algorithm for the treatment of intertrochanteric fractures based on the results known from the literature. The following questions will be addressed:

- Treatment in the case of polytrauma or monotrauma;
- Reconstruction vs. arthroplasty;
- Open vs. closed reduction;
- Reconstruction with or without additional cables and plates;
- Cephalomedullary nail vs. dynamic hip screw (DHS);
- Long cephalomedullary nail vs. short cephalomedullary nail.

## 2. Methods

A literature search was performed by the authors in orientation to the PRISMA.

**Figure 1:**
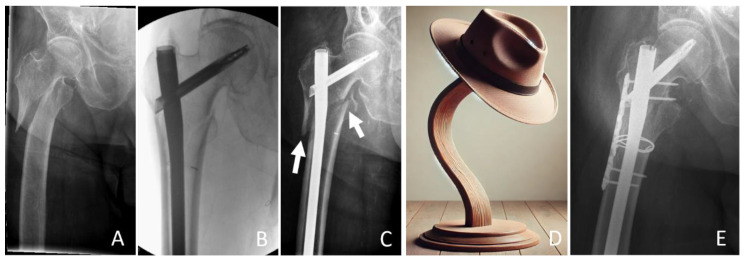
Case of an unstable proximal femur fracture. (**A**) Preoperative, (**B**): intraoperative image after reconstruction via a long cephalomedullary nail, (**C**): early postoperative secondary dislocation. (**D**): Illustration of the “hat rack instability”. (**E**): Postoperative image after revision using additive plates and cables. Arrows: Indicating fracture dislocation.

- Regulations (Preferred Reporting Items for Systematic review and MetaAnalysis); however, the focus was rather on examining and discussing controversies. Initially, the title and abstract were screened, and, if applicable, the whole manuscript was screened. The following search terms were used in PubMed/MEDLINE in August 2024: (intertrochanteric fracture) AND (long cephalomedullary nail) OR (short cephalomedullary nail); (intertrochanteric fracture) AND (open) OR (closed reduction), (intertrochanteric fracture) AND (arthroplasty), (intertrochanteric fracture) AND (dynamic hip screw) OR (gliding hip screw), (intertrochanteric fracture) AND (polytrauma), (intertrochanteric fracture) AND (implant failure). Data extraction was focused on literature, including manuscripts from the last decade. Eligibility criteria were (1) manuscripts in the English or German language, (2) clinical or biomechanical studies reporting the outcome after treatment of intertrochanteric fractures, and (3) data published in a peer review process. Studies were excluded if they were conference papers, letters or comments, or case reports. A ranking of the level of evidence was performed, favoring manuscripts in a hierarchical process with (1) high-quality randomized trials or prospective studies, (2) prospective comparative studies, (3) case–control studies, and (4) case series. A low level of evidence is interpreted as a limitation, which is discussed if applicable in the manuscript.
- Only for Figure 1D was AI (ChatGPT) used to produce the image. AI was not further used in the processing process of this work. Neither in the literature research process nor in the writing or processing process of this manuscript was AI used.

## 3. Classification

The most common classification in clinical practice is that according to the AO/OTA, with increasing complexity [6]. A1 fractures are generally stable fractures, in which the medial fracture line allows direct support. They are classified as A1.1, with a fracture line through the intertrochanteric line, A1.2, through the greater trochanter, and A1.3, distal to the lesser trochanter. The fractures are not multifragmentary. With regard to A1.3 fractures, however, there is already a loss in primary stability for axial loading. A2 injuries are multifragmentary, with A2.1 fractures involving a fractured lesser trochanter fragment, A2.2 a multifragmentary greater trochanter fragment, and A2.3 fractures a medial fracture extension > 1 cm distal to the lesser trochanter. A3 fractures are unstable intertrochanteric fractures, including the A3.1 fractures as so-called reverse oblique fractures, the A3.2 fractures, described as transverse fractures, which have a certain primary stability, and the unstable A3.3 fractures, in which a further lesser trochanteric fragment exists.

In addition, the Evans classification does not differentiate morphotypes but rather differentiates the primary stability after reduction as stable, unstable, or, as a special form, reverse obliquity fractures with primary instability. The focus here is on the reconstructability of a medial support, which describes the degree of stability after reduction [7,8]. 

## 4. Monotrauma or Polytrauma

**Figure 2:**
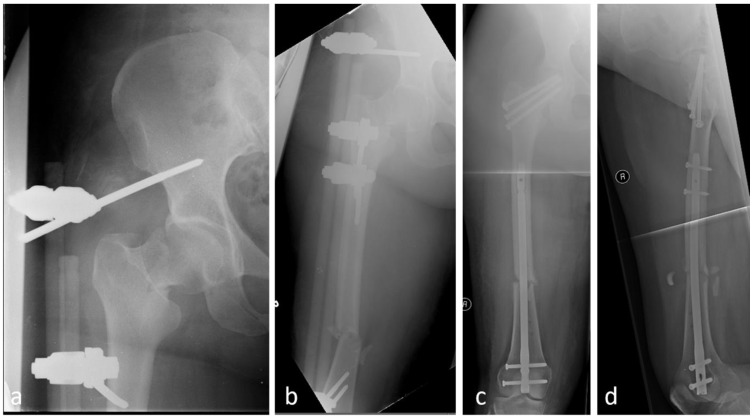
Treatment of a combined femoral neck fracture and femoral shaft fracture in a polytraumatized patient via a pelvifemoral external fixator (**a**,**b**) and final treatment via screws and retrograde nail after initial stabilization, (**c,d)**: final stabilization in ap and lateral imaging with cannulated strews and retrograde nail.

As the majority of proximal femoral fractures are observed in geriatric patients as part of a low-energy trauma, the standard treatment concept is to provide prompt surgical treatment in order to avoid secondary complications and reduce mortality [9]. Furthermore, geriatric comanagement is essential to realize prompt and appropriate treatment [10]. However, if the patient is polytraumatized, with serial injuries to the lower extremities, more general treatment strategies must be considered. For example, treatment options are possible within the scope of the Damage Control Strategy (DCO) or using Early Total Care concepts (ETC). The injury pattern, signs of shock, and the patient’s overall constitution are crucial here. In the case of serial injuries according to the DCO, a study group from Murnau have described treatment of a femoral neck fracture using DHS and additional treatment of a shaft fracture using an external fixator. In a second operation, this is converted to a retrograde femoral nail. In the case of a stable patient, according to the ETC strategy, in a single initial operation the treatment strategy could be a reconstruction via a long cephalomedullary nail [11]. However, in intertrochanteric fractures, which need to be treated via a cephalomedullary nail, this strategy does not apply. An alternative option is a pelvifemoral external fixator, which is easy to use and a suitable tool for serial injuries of the femur in the realm of DCO [12] (Figure 2).

### Fixation vs. Arthroplasty

When treating proximal femoral fractures, the aim is to achieve mobilization under full weight bearing in order to avoid further complications. This goal must be achieved by incorporating the least possible surgical trauma. Although the treatment strategy for type 31-B fractures according to the AO/OTA, i.e., femoral neck fractures in older people, using arthroplasty is generally established, this does not apply to intertrochanteric fractures. However, recent studies have given rise to a discussion about using arthroplasty in a narrow range of indications for intertrochanteric fractures. For A1 and A2 fractures, Lu X et al. were able to demonstrate that the surgical trauma is larger, the operating time is longer, and the blood loss is greater, but the function and especially the mortality are the same [13]. Therefore, we ask whether fractures that do not allow primary full weight bearing, e.g., due to morphology, osteoporosis, or patient compliance, should be treated via arthroplasty. With regard to unstable fractures and, in particular, A3 fractures, Sniderman J. et al. were able to show that open reconstruction is associated with more blood loss, a higher revision rate, and poorer mobility compared to arthroplasty [14]. Although the strength of the study is the design, as a matched cohort study with 150 patients in each group, the retrospective nature and the missing patient reported outcome parameters are a limitation. Nevertheless, an existing dogma that A3 fractures should be treated via a long nail must be critically reconsidered and reevaluated.

## 5. Different Forms of Reconstruction

### 5.1. Basic Considerations

There is still a controversial discussion regarding the stability of the screw or blade in the femoral neck. Initially, it was assumed that a screw position as close to the calcar as possible would provide the greatest stability. This assumption was based on a particularly dense arrangement of the pressure trabeculae and thus good anchoring in this area. In 1995, Baumgaertner published his work on the relevance of the tip–apex distance (TAD) [15]. He was able to show that the failure rate was lower with a tip–apex distance < 25 mm. Although these data are still relevant and should be taken into account, more recent studies also show that a center–center position shows greater stability and a lower failure rate than a screw position outside the center position [16,17,18]. However, since the individual varus/valgus angle of the femoral neck is not always congruent with that of the prefabricated nails, deviations can occur that do not allow for congruent positioning in the center–center position. Thus, both assumptions, which have proven their correctness both biomechanically and in clinical studies, are not contradictory, but should be considered similarly.

Further aspects of implant failure, like the cut-out, are the choice of neck implant and patient-specific properties like osteoporosis. Both have been attempted to be addressed via the usage of either screws or helical blades. Stern et al. showed, in a prospective randomized study, no advantage of one of the implants with regard to cut-out rate, but once again observes the TAD to be the most relevant factor [19,20]. However, in a retrospective analysis of 362 patients, the cut-out rate was significantly higher in patients treated via a helical blade compared to a lag screw [21]. Further studies seem to show a trend towards higher failure rates of helical blades in combination with an increased TAD compared to compression screws [22,23]. One option, especially in osteoporotic bone, is cement augmentation, which is a safe method and leads to good functional results [24]. Besides PMMA, augmentation with calcium phosphate or other biological augmentation materials can be used. The controversy about PMMA is the potential disturbance of the bone metabolism and the potential risk for cartilage damage due to heat production. However, so far these risk factors have not proven to be clinically relevant, which favors PMMA due to the direct initial stability. Furthermore, in another prospective randomized study, Kammerlander et al. showed a trend towards lower revision rates in the case of cement augmentation [25]. Finally, as a more stable fixation of the femoral head screw seems favorable, Serrano et al. asked whether two screws are more stable than one screw and observed, in a retrospective review of 413 patients, significantly higher failure rates in patients who were treated via a single screw [26]. However, Berger-Groch et al. observed in a prospective randomized trial no difference between the two therapeutic options [27].

### 5.2. Open vs. Closed Procedure

With regard to primary stability, anatomical reduction is of outstanding importance. If this is not possible via a closed reduction, according to the fracture morphology, one has to clarify whether primary stability can be achieved through open anatomical reduction. Furthermore, stable fractures can occasionally be irreducible due to interposition of the fragment behind the psoas tendon and require open reduction [28,29]. Unfortunately, imprecise wording and understanding of open or closed procedures in the literature complicate the comparability of the studies. Generally, minimally invasive reduction techniques such as collinear clamp, ball spike pusher, bone hook, or a minimal invasive cerclage wire are considered to be closed procedures. However, in the case of unstable fractures, which are not reducible via a closed reduction technique, not only open reduction but also arthroplasty need to be discussed. As mentioned above, Sniderman et al. were able to show that using arthroplasty was associated with less blood loss and a lower revision rate than open reduction and reconstruction. The researchers were also able to demonstrate faster mobilization with regard to clinical outcome parameters [14].

### 5.3. Reconstruction With or Without Additional Cables and Plates

**Figure 3:**
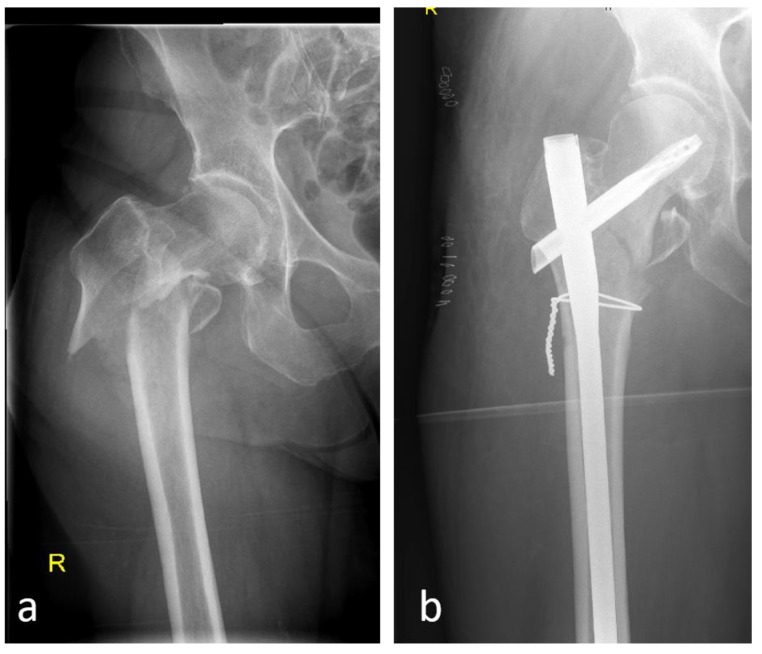
Initial 31-A3 fracture with dislocation of both fragments (**a**,**b**) after reduction in lateral position, cable fixation via minimally invasive surgery and fixation via a long cephalomedullary nail.

If primary stability in unstable fractures cannot be achieved sufficiently by means reconstruction via a cephalomedullary nail, the usage of additional cables or plates should be considered (Figure 3). The relevance for the blood supply is often controversially discussed; however, so far only one study using a rabbit model has been able to demonstrate a relevant reduction in periosteal blood supply by cables [30]. If the decision is made in favor of open reduction, cables have been shown to generate great pullout stability. Plates, whether medial or lateral, have also been shown to provide additional stability, either as a buttress plate or as a tension band plate [31,32].

### 5.4. Cephalomedullary Nail vs. Dynamic Hip Screw (DHS)

With the beginning of the new century, a trend towards a significant decline in the usage of DHSs has been observed compared to the usage of cephalomedullary nails among orthopedic surgeons in the United States. It is argued that surgeons believe that cephalomedullary nails are easier and associated with improved outcomes, or are biomechanically superior to a DHS [33,34].

In a study by Kyriakopoulos et al., who biomechanically investigated the primary stability of A1 and A2 fractures between the two treatment methods, no difference in stable fractures, but a clear advantage of reconstruction via a cephalomedullary nail in unstable fractures, could be seen [35]. Weiser et al. also examined the primary stability of A2.3 fractures and observed a significantly better stability in reconstruction via a cephalomedullary nail (8480.8 ± 1238.9 N) than in reconstructions via a DHS (2778.2 ± 196.8 N) [36]. However, it is noticeable that the axial load of around 2800 N, which is applied until implant failure of the DHS is observed, corresponds to the fall of a 70 kg patient from a height of 4 m or a 280 kg patient from a fall height of 1 m.

With regard to the clinical function and complication rates, although reconstruction via a cephalomedullary nail is more stable compared to a two-hole DHS, the relevance of gluteal muscle injury via the entry point remains controversial. Especially in geriatric patients, we aim to preserve as much functional gluteal muscle as possible.

In a prospective randomized controlled trial, Reindl et al. did not observe a difference between the two procedures with regard to outcome parameters [37].

However, in a systematic review, Zeelenberg et al. asked for the outcome of unstable A2 fractures, treated with one of the two methods, and observed in patients treated via a cephalomedullary nail a better function, measured in functional scores such as the HSS and the Parker Mobility Score, shorter time to full weight bearing, a lower rate of implant failure, and a lower rate of pseudoarthrosis and leg length discrepancies [38]. Especially with regard to stable fractures, treatment with a DHS may still be considered, despite the lower primary stability compared to reconstruction via a cephalomedullary nail.

### 5.5. Long Cephalomedullary Nail vs. Short Cephalomedullary Nail

Generally, comparing long and short cephalomedullary nails with regard to the clinical outcome shows that using long cephalomedullary nails results in a longer operating time and a longer interval until pain relief is achieved. [39,40]. There is certainly a bias in the fact that more complex fractures are more likely to be treated with a long cephalomedullary nail and that 1. the reduction, nail insertion, possible additional drilling of the medullary cavity, and distal locking prolong the operating time and 2. additional open reduction techniques and the complexity of the fracture are associated with delayed healing and a longer postoperative pain interval. With regard to the complication rate for unstable fractures, no significant difference is described in the literature. This is particularly interesting because stabilization via the entire length of the femur using a long nail is supposed to reduce the risk of secondary dislocation, peri-implant fracture, or implant failure [40,41,42]. Finally, all controversial arguments culminate in the correct treatment of 31-A3 fractures. Whereas Irgit et al. state that long cephalomedullary nails are the preferred treatment method due to low reoperation rates and high rates of healing [43], Linhart et al. in 2023 were unable to determine any difference in the stability of the two types of treatment based on biomechanical studies [44]. Furthermore, Shannon et al. proved in a randomized prospective study that short nails accommodate a subrochanteric fracture extension of 3 cm [45]. The additional, subjectively perceived stability and thus greater safety when treating 31-A3 fractures using a long cephalomedullary nail must be reconsidered and reviewed in the future in order to be able to avoid the above-mentioned disadvantages if necessary. Due to the missing large prospective randomized controlled trials, a profound risk analysis of possible higher revision rates is not possible. So far, a cost–benefit analysis comparing long and short cephalomedullary nails would be of interest, but is not applicable, either.

### 5.6. Factors Defining Stability

The stability of the fracture is defined by the medial support. If this is possible, primary stability can be assumed, which also allows full weight bearing. The thickness of the lateral wall fragment defines a secure anchoring distance of the implant against varus stress. A thickness of >18.55 mm has been shown to be superior in terms of secondary dislocation [46]. The relevance of the distance between the lateral distal fracture extension and the distal locking bolt of the nail for secure intramedullary support against varus stress remains unclear. The influence of any metaphyseal comminution zone on fracture healing and thus definitive stability also remains unclear. These fractures require more attention in the future, as the femoral head suffers from great instability like a hat on a hat rack. We call this “hat rack instability”, which requires increased attention in order to be treated adequately (Figure 1).

### 5.7. Arthroplasty

As shown by Sniderman et al., arthroplasty can be relevant in selected cases [14]. In these cases, the refixation of the greater trochanter seems to be less relevant. Since the treatment of intertrochanteric fractures via arthroplasty is not the gold standard, only limited data exist to interpret the outcome, and other general considerations need to be extrapolated, for instance, in the case of femoral neck fractures treated via arthroplasty. The German registry data show that there are failure probabilities of 4% for primary total hip arthroplasty (THA), 5% for treatments with bipolar hip arthroplasty (BHA), and 8% for non-elective THA (EPRD Annual Report 2023). With regard to the question of cementation, Finnish, English, Australian, and Norwegian registry data show the superiority of cementation in terms of revision rates [47,48,49,50,51]. Considering the acetabular treatment options in THA in a fracture situation, in recent years, the usage of bipolar cups due to lower dislocation rates has emerged. In Germany, the numbers have tripled since 2014. Especially in geriatric patients, there is an increasing number of double mobility cups showing lower dislocation and revision rates. Although operation time is longer and the surgical trauma is bigger compared to treatment with BHA, the dislocation rate and mortality appear to be lower. A major limitation is a bias in patient selection and general indication [52].

### 5.8. Algorithm for Treating Intertrochanteric Femoral Fractures

**Figure 4:**
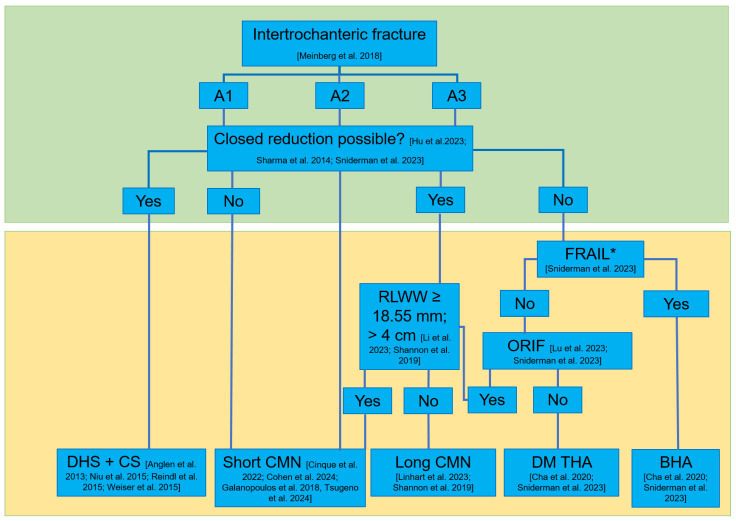
Decision algorithm for the treatment of intertrochanteric femoral fractures. ORIF: open reduction and internal fixation, RLWW: residual lateral wall width, LFA: lateral fracture distance, DHS ± CS: dynamic hip screw and compression screw, DM THA: double mobility total hip arthroplasty, CMN: cephalomedullary nail, BHA: bipolar hip arthroplasty. *: Besides the fact of being frail, this summarizes a complex clinical situation including overall health, bone quality, and the specific needs of each individual.

Several aspects mentioned above should be considered in the therapeutic decision tree. The authors are aware of the controversies arising from the algorithm. However, we intend to use these controversies from the literature to highlight the lack of sufficient data for a sufficient treatment algorithm and intend to debate one suitable option (Figure 4). The presented algorithm does not reflect the institutional decision tree but depicts relevant aspects that are included in our clinical decision-making. Both the interests of minimal invasiveness and maximum stability to achieve direct full weight bearing occasionally compete with one another. Reconstruction with a DHS with or without a compression screw can be recommended for stable A1 fractures in the case of anatomical repositioning. If anatomical repositioning is not achievable, the cephalomedullary nail is advantageous. For A2 fractures, treatment using a short intramedullary nail can generally be recommended. For A3 fractures, in addition to anatomical repositioning, the fracture morphology is also crucial for appropriate primary stability. Fractures with a sufficient lateral wall of >18.55 mm and a sufficient distance of >4 cm between the lateral fracture extension and the distal locking seem to be stable and safe using a short intramedullary nail. If this fracture morphology is not present, treatment using a long intramedullary nail should be used to ensure stability until this question has been investigated in more detail in the literature. If closed reduction of A3 fractures is not anatomically possible, patient-specific factors should be taken into account again. Especially since the literature shows that arthroplasty is less traumatic than open reduction and enables direct full weight bearing, treatment using BHA can be considered. A suitable differentiation between reconstruction and arthroplasty has so far been made in the literature between under and above 90 years. Further work must certainly create a more precise differentiation of the patient collective. The allocation to the correct treatment is the responsibility of the surgeon and must be made in consultation with the patient. If reconstruction is not reasonable, treatment using cemented THA with a double mobility cup can be considered. However, if open reduction is anatomically possible, the above-mentioned fracture-specific properties remain relevant to differentiate between treatment using a long or short proximal femoral nail. In summary, the recommendation of arthroplasty in a patient with an intertrochanteric fracture needs to be reviewed carefully. There is no evidence for a general recommendation of this procedure. However, there is some evidence that in certain situations arthroplasty might be a suitable option, taking into consideration multiple aspects involving overall health, bone quality, and the specific needs of each individual. We are aware of the controversy of this algorithm and expect a critical interpretation of several aspects. This manuscript is not intended to promote hazardous procedures but rather to highlight these controversies, which need further high-level prospective, randomized controlled trials to further clarify the suitability of these therapeutic options.

## 6. Conclusions

In an aging population, intertrochanteric fractures become more relevant. Established algorithms give good guidelines for therapeutic options. However, new data show that these guidelines should be interpreted carefully, and individual therapeutic options should be considered. In particular, further morphological properties that describe the primary stability of intertrochanteric fractures need to be clarified. In the case of instable fractures, arthroplasty can be considered as a therapeutic option. Larger and more well-conducted, high-quality trials are needed in order to gain more secure answers.

## Data Availability

There are no further data available.
